# Metabolic Pathway Analysis: Advantages and Pitfalls for the Functional Interpretation of Metabolomics and Lipidomics Data

**DOI:** 10.3390/biom13020244

**Published:** 2023-01-27

**Authors:** Sofia Tsouka, Mojgan Masoodi

**Affiliations:** Institute of Clinical Chemistry, Inselspital, Bern University Hospital, 3010 Bern, Switzerland

**Keywords:** metabolomics, metabolism, pathway analysis, over-representation analysis, network topology

## Abstract

Over the past decades, pathway analysis has become one of the most commonly used approaches for the functional interpretation of metabolomics data. Although the approach is widely used, it is not well standardized and the impact of different methodologies on the functional outcome is not well understood. Using four publicly available datasets, we investigated two main aspects of topological pathway analysis, namely the consideration of non-human native enzymatic reactions (e.g., from microbiota) and the interconnectivity of individual pathways. The exclusion of non-human native reactions led to detached and poorly represented reaction networks and to loss of information. The consideration of connectivity between pathways led to better emphasis of certain central metabolites in the network; however, it occasionally overemphasized the hub compounds. We proposed and examined a penalization scheme to diminish the effect of such compounds in the pathway evaluation. In order to compare and assess the results between different methodologies, we also performed over-representation analysis of the same datasets. We believe that our findings will raise awareness on both the capabilities and shortcomings of the currently used pathway analysis practices in metabolomics. Additionally, it will provide insights on various methodologies and strategies that should be considered for the analysis and interpretation of metabolomics data.

## 1. Introduction

Over the last decades, the importance of robust computational pipelines to interpret experimental data has increased significantly. This is, at least in part, due to continuous advancements in analytical techniques that allow us to capture a large number of metabolites. Although various tools have emerged for the analysis of metabolomic data, proper guidelines and consensus in their correct use and interpretation is still lacking [1,2].

Pathway analysis aims to link changes in metabolic compounds to biological pathways [3]. Over-representation (or enrichment) analysis (ORA) [4,5,6,7] is one of the most commonly used methods in pathway analysis. The various statistical techniques used by ORA do not take into account the measured fold changes, considering only the number of statistically significant metabolites using arbitrary thresholds such as p-value which results in a loss of potentially valuable information or in pseudo-positive outcomes. Other approaches include functional class scoring (FCS), commonly used by gene set enrichment analysis (GSEA) [8,9] as well as tools that have incorporated network topological properties [10,11,12,13,14]. Topological pathway analysis (TPA) is based on the conversion of the metabolic network to a series of graphs, and the subsequent scoring of the associated pathway impact through various measures [15]. This approach has been used to study various pathophysiological conditions, such as non-alcoholic steatohepatitis (NASH) [16], Alzheimer’s [17], and cancer [18].

The study of metabolism in the frame of graph theory has been around for years and can uncover several structural features of the metabolic network [19,20,21,22]. The most common graphical representation of metabolic networks considers the vertices (or nodes) in the network as metabolites and the connecting edge between any two vertices as a reaction [15,23,24]. Other common graphical representations of metabolism are bipartite graphs, where vertices represent metabolites and metabolic reactions (or the enzymes that catalyze them), and edges link the metabolites to the reactions in which they participate [25,26], and undirected graphs where vertices represent metabolic reactions and joining edges signify a shared participating metabolite [27,28].

Traditionally, ORA and TPA utilize publicly available pathway collections such as KEGG [29,30], Reactome [31,32], and Biocyc [33], which are inherently different in pathway definitions and compound identifiers and can affect the quality of results for these two approaches. Apart from these inherent differences, other factors can greatly affect the quality of results for these two methods. For example, it is common that each of the pathways is evaluated separately from the rest, without considering the connectivity with other pathways of the metabolic network. Moreover, the connectivity between different metabolic pathways and centrality of metabolites participating in multiple pathways have not been fully investigated. Another important issue in pathway definition is the consideration of metabolic reactions that are catalyzed by non-human native enzymes. Such reactions are associated with microbiota and they play a part in the metabolome phenotype of the organism. Even though the impact of connectivity and the inclusion of non-human native reactions have not been investigated in the past, TPA is still one of the leading methods used for the functional analysis of metabolomics data. Despite the existence of user-friendly software, it is important to be mindful of the methodology best suited to any given dataset, and the interpretation of the functional outcome. In this manuscript we aim to investigate the effects of connectivity and inclusion of non-human native reactions in TPA and to provide insight on the interpretation of the functional outcome. Using publicly available datasets that cover a wide spectrum of conditions, we examine and discuss how pathway definition and connectivity affect the results of TPA. Additionally, we compare ORA results with TPA. We believe that our findings will raise some awareness on both the capabilities and shortcomings of these commonly used methods and will encourage further research on their proper usage and interpretation.

## 2. Materials and Methods

To investigate the effect of connectivity and inclusion of non-human native reactions, we performed TPA on four publicly available metabolomics datasets (Table 1). We used the KEGG pathway database [29,30] for pathway definitions. In order to compare and evaluate the results between different methodologies, we also performed ORA on the same datasets. The statistically significant compounds for each dataset were matched to their KEGG identifiers where possible (Table 1). In the absence of a specific identifier, the matching of compounds was performed using in-house tools and manual curation. We also used an online tool (MetaboAnalyst’s compound ID conversion) [34] to match the same datasets to KEGG IDs and showed the difference in the successfully mapped compounds (Table 1). We provide the datasets and identifiers we used in Table S1.

### 2.1. Graph Theory Concepts and Metrics

Metabolic networks can be translated to graphs, where each node represents a metabolite and each edge represents a reaction. In turn, a graph can be mathematically represented through an adjacency matrix, A. A is a square matrix, whose elements indicate whether pairs of nodes are adjacent or not in the graph. Since metabolic reactions do not always have the capability to operate in both directions, we considered a directed graph for our analysis, which means that A is not symmetric. For the case of non-single-substrate–single-product reactions, the reaction was split into pairwise singular ones. For example, reaction A+B→C+D would be split in the elementary reactions A→C, A→D, B→C, and B→D. Additionally, the weight of each edge was considered to be equal to the number of enzymes capable of catalyzing the associated reaction, i.e., through isoenzymes or different cofactor pairs. 

Vertex centrality measures express the importance of a node (or vertex) in the graph network and provides information about the latter’s layout. Various well-established measures of centrality have been defined based on degree, closeness, and betweenness criteria [39,40]. For biological networks, betweenness centrality [41] is commonly used, and measures how often a certain node appears on paths connecting other nodes, thus providing a very relevant biological expression of importance. The scaled betweenness centrality of a node v in a directed graph is calculated as:

$$BC\left(v\right)=\frac{\sum_{a\neq v\neq b}\frac{\sigma_{ab}\left(v\right)}{\sigma_{ab}}}{\left(N-1\right)\left(N-2\right)}$$

where $\sigma_{ab}$ is the total number of shortest paths connecting nodes a and b, $\sigma_{ab}\left(v\right)$ is subset of them that pass through node v, and N is the total number of nodes.

### 2.2. Pathway Connectivity and Human/Non-Human Native Considerations

We explored the effect of including non-human-specific reactions in the metabolic pathway definitions. In the KEGG database these pathways are labelled as “reference” (or “generic”), as opposed to the human-only pathway designations (“organism-specific”). Hence, in this manuscript we refer to them as “generic” and “human-only”, respectively. 

Additionally, we investigated the effect of pathway connectivity on TPA. The two approaches of disconnected and connected pathways are referred to as “disconnected” and “connected” in this manuscript, respectively. In the disconnected approach, each pathway was considered independent of the others, and the centrality scores were calculated accordingly using the above formulas. In the connected approach, all the connections between pathways were taken into account prior to the calculation of the centrality scores.

### 2.3. TPA Impact Score Calculation

For each pathway, the impact score was calculated as:

$$Impact=\overset{w}{\underset{i=1}{\sum }}BC_{i}/\overset{W}{\underset{j=1}{\sum }}BC_{j}$$

where W and w are the number of total and statistically significant compounds within the pathway, respectively, and BC is the betweenness centrality score of the compound.

### 2.4. ORA Probability Calculation

There are two tests that are most commonly used in ORA, namely the hypergeometric and Fisher’s exact tests. Both tests are based on the hypergeometric distribution, which describes the discreet probability of *k* successes in m random draws without replacement, from a population of total size *M* that contains *K* objects with that attribute. We chose to use the hypergeometric test for our analysis.

In terms of metabolomics evaluation and for a single pathway *i*, *M* is the total number of metabolites in all pathways of the collection, *K* is the number of metabolites in pathway *i*, *m* is the number of compounds measured in the experiment, and *k* is the subset of m that belongs to pathway *i*. The probability for the over-representation of each pathway was calculated as:

$$p\left(k\right)=\frac{\left(\begin{array}{c} K\\ k \end{array}\right)\left(\begin{array}{c} M-K\\ m-k \end{array}\right)}{\left(\begin{array}{c} M\\ m \end{array}\right)}$$

where $\left(\begin{array}{c} i\\ j \end{array}\right)$ is the binomial coefficient.

### 2.5. Hyper Parameter Hub Penalization Scheme

One of the challenges in the connected approach is the appearance of hubs in the metabolic graph. Hubs are nodes that are very central in the metabolic network, which results in high centrality scores for these nodes compared to the vast majority of other nodes within the metabolic network. Thus, their presence might bias the scoring of pathways in a non-realistic and unbalanced manner [24,42,43]. To address this, we employed a penalization scheme for hubs in order to moderate their effect. The method we used is a one-sided penalized median formulation, which effectively moderates outlier node scores according to the formulation:

$$BC_{penalized}=\{\begin{array}{c} BC\left(\frac{2d_{med}}{BC-\tilde{BC}}\right),\;if\;BC>\tilde{BC}+2d_{med}\\ \frac{BC^{2}}{BC+\frac{d_{med}{}^{2}}{BC-\tilde{BC}}},\;if\;BC>\tilde{BC}+d_{med} \end{array}$$

where BC is the betweenness centrality score of a compound, $\tilde{BC}$ is the betweenness centrality score population median, and $d_{med}$ is the betweenness centrality score population median average deviation (MAD), which is defined as:

$$d_{med}=\frac{1}{N}\overset{n}{\underset{i=1}{\sum }}\left|BC_{i}-\tilde{BC}\right|$$

As can be seen in the formula, outlier centrality scores that exceed two MADs are reduced to within one and two MADs, and ones that exceed one MAD are reduced to within the median and one MAD. This method of moderation was applied only in cases of connected approach scoring as stated in the results section, and the median and MAD values were calculated within each pathway. In the extreme case where both the median and MAD values of a single pathway where equal to zero, the MAD value was set to $10^{-6}$.

### 2.6. Software

We developed an R-based in-house software. All calculations and figures were made using R version 4.0.2. Pathway definitions were obtained from KEGG KGMLs (September 2021). Betweenness centrality scores were calculated using the betweenness() function of the igraph package.

## 3. Results

### 3.1. The Functional Importance of Valine and Tryptophan Biosynthesis Is Highly Impacted by Consideration of Non-Human Enzymes

**Figure 1:**
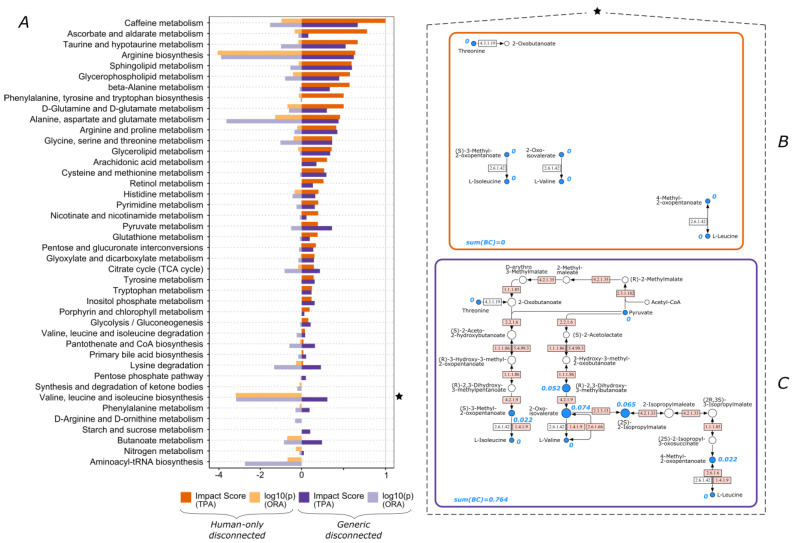
Pathway scoring comparison for dataset 1a. (**A**) TPA impact scores and ORA log10(p) values for all pathways (pathways exhibiting zero values in all cases are omitted). ★ “Valine, leucine and isoleucine biosynthesis” pathway for (**B**) human-only disconnected and (**C**) generic disconnected. Blue color denotes significant compounds in this dataset, blue values are the computed betweenness centrality scores for these nodes, sum(BC) is the sum of betweenness centrality scores for all the nodes of the pathway, and node size corresponds to the relevant betweenness centrality scores of the nodes within the pathway. White and pink boxes denote human native and non-human native enzymatic reactions, respectively.

One of the challenges we face in human metabolic phenotyping is the consideration of metabolic reactions that are catalyzed by non-human native enzymes, usually associated with microbiota. Within this study, we investigated the impact of inclusion of non-human native enzymes on the outcome of TPA. We observed that certain human-only pathways cannot have an impact score larger than zero, independent of the dataset, i.e., they never appear as significantly impacted. This is due to multiple isolated and disconnected reactions within the pathway. In this case, all of the participating metabolites have a calculated betweenness centrality score of zero. However, this issue was resolved when non-human enzymes are taken into consideration in the network and all of the defined pathways are well connected within themselves. For example, “Valine, leucine and isoleucine biosynthesis” pathway consists of four individual reactions that are catalyzed by human native enzymes (Figure 1B). The corresponding generic pathway that includes enzymatic reactions catalyzed by non-human enzymes, allows for the proper calculation of centrality scores due to the additional inclusion of multiple connecting reactions (Figure 1C). For example, for dataset 1a, the calculated impact score for “Valine, leucine and isoleucine biosynthesis” pathway increased from zero to 0.31 (Figure 1A). Specifically, 2-oxoisovalerate became one of the most highly scored compounds in the pathway, which leads to a major contribution to the impact score. The drawback of the human-only approach is particularly evident in this example where the pathway impact score remained zero even though seven out of eight total participating compounds were significantly different (Figure 1B).

On the other hand, the generic pathway offers another major advantage. In this case, multiple metabolites cannot be “accessed” by native human enzymes, thus getting lost in a human-only definition. For example, in Figure 1C two additional compounds were considered in the generic impact score calculation, namely pyruvate and (2S)-2-isopropylmalate. The latter contributed significantly to the impact score of the pathway, since it is one of the most central nodes of the pathway and thus has a high betweenness centrality score. This is in agreement with our previous observation in another COVID-19 study [44].

**Figure 2:**
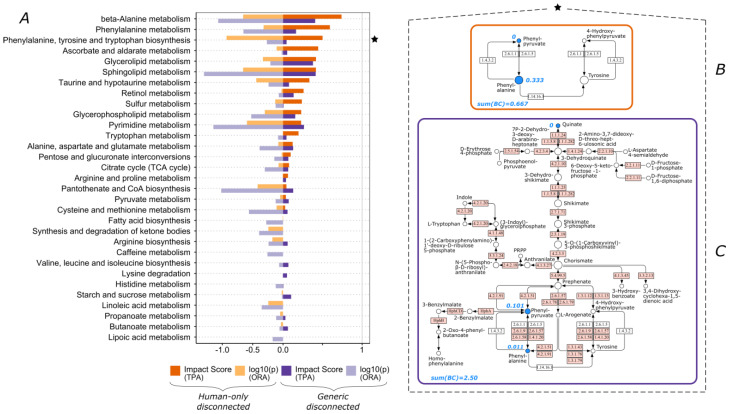
Pathway scoring comparison for dataset 1b. (**A**) TPA impact scores and ORA log10(p) values for all pathways (pathways exhibiting zero values in all cases are omitted). ★ “Phenylalanine, tyrosine and tryptophan biosynthesis” pathway for (**B**) human-only disconnected and (**C**) generic disconnected. Blue color denotes significant compounds in this dataset, blue values are the computed betweenness centrality scores for these nodes, sum(BC) is the sum of betweenness centrality scores for all the nodes of the pathway, and node size corresponds to the relevant betweenness centrality scores of the nodes within the pathway. White and pink boxes denote human native and non-human native enzymatic reactions, respectively.

Alternatively, the generic approach can lead to much lower pathway impact scores compared to the corresponding human-only one. This occurs if the human-only reactions are well connected within the pathway with limited number of metabolites. Such is the case of the “Phenylalanine, tyrosine and tryptophan biosynthesis” pathway, as its score decreased from 0.5 to 0.04 for dataset 1b (Figure 2). The human-only pathway encompasses just four metabolites compared to thirty-four in its generic pathway. Similarly, the percentage of reactions contained in the human-only versus the generic is about 12%. For dataset 1b, the number of participating statistically significant compounds was two for the human-only pathway, and it increased to three for the generic pathway (Figure 2B,C). Due to a remarkable difference in the ratio of significantly changed metabolites to the total metabolites (generic / human-only), the impact score between the two pathways was significantly different, and thus further highlights the impact of pathway size and definition on the outcome. In the human-only case, 50% of the participating compounds were altered, leading to a high impact score. However, in the generic case, this percentage was reduced to about 9%.

**Figure 3:**
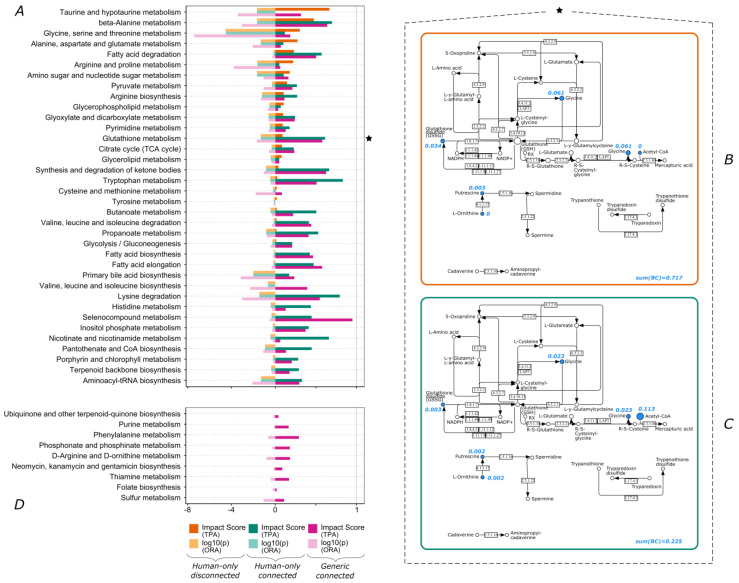
Pathway scoring comparison for dataset 2. (**A**) TPA impact scores and ORA log10(p) values for all pathways (pathways exhibiting zero values in all cases are omitted). ★ “Glutathione metabolism” pathway for (**B**) human-only disconnected and (**C**) human-only connected. Blue color denotes significant compounds in this dataset, blue values are the computed betweenness centrality scores for these nodes, sum(BC) is the sum of betweenness centrality scores for all the nodes of the pathway, and node size corresponds to the relevant betweenness centrality scores of the nodes within the pathway. (**D**) TPA impact scores and ORA log10(p) values for microbiome related pathways.

We had a similar observation for dataset 2, which focuses on microbiota metabolism (Figure 3). Inclusion of non-human native enzymes in pathway definitions led to a nonzero score for several pathways (Figure 3D). There are several studies reporting the contribution of human microbiome to many of these pathways, such as “Sulphur metabolism” [45], “Phenylalanine metabolism” [46], “D-arginine and D-ornithine metabolism” [47], and “Folate biosynthesis” [48]. In this case, these pathways were captured only in the generic approach and not in the human-only one. It is worth mentioning that the non-native enzymes do not exclusively belong to the microbiome. Some of them might still represent enzymes present in other organisms such as fungi or archaea, or enzymes that do exist in humans but have not been identified in the human genome. 

### 3.2. Lipid Metabolism and Amino Acid Metabolism Pathway Are Highly Impacted by Connectivity

**Figure 4:**
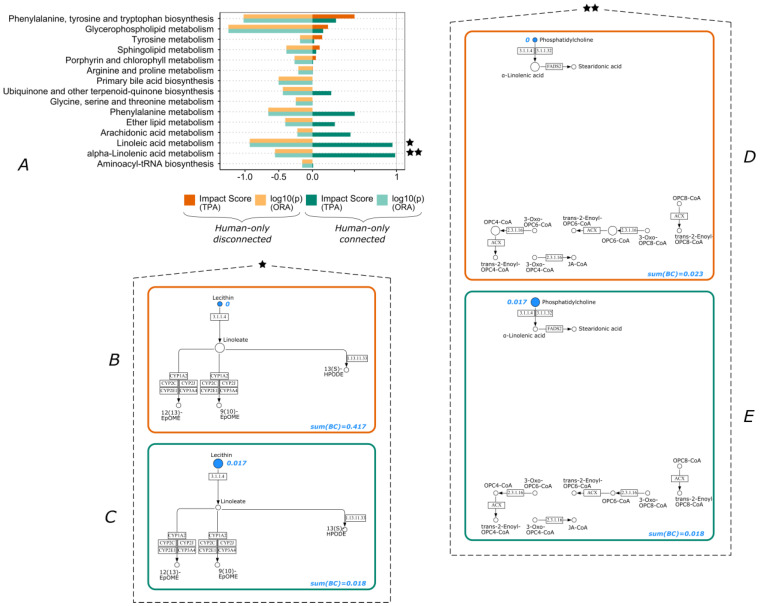
Pathway scoring comparison for dataset 3. (**A**) TPA impact scores and ORA log10(p) values for all pathways (pathways exhibiting zero values in all cases are omitted). ★ “Linoleic acid metabolism” pathway for (**B**) human-only disconnected and (**C**) human-only connected. ★★ “alpha-Linolenic acid metabolism” pathway for (**D**) human-only disconnected and (**E**) human-only connected. Blue color denotes significant compounds in this dataset, blue values are the computed betweenness centrality scores for these nodes, sum(BC) is the sum of betweenness centrality scores for all the nodes of the pathway, and node size corresponds to the relevant betweenness centrality scores of the nodes within the pathway.

Although metabolic pathways are highly interconnected and affected by each other, many TPA tools consider each pathway as its own isolated network. This can result in major differences in pathway scoring. Even a single metabolite can have an extremely different centrality score when accounting for connectivity of pathways. For example, we observed this effect with dataset 3 for the “Linoleic acid metabolism” and “alpha-Linolenic acid metabolism” pathways. Their respective impact scores changed from zero for the disconnected approach to almost 1 for the connected approach (Figure 4B,C and Figure 4D,E, respectively). The difference was so evident in these pathways because the participating compounds are not connected very well with the rest of the metabolic network, with the exception of lecithin and phosphatidylcholine (Figure 4B,C, respectively). This effect is more apparent in “Glutathione metabolism” pathway for dataset 2 (Figure 3). In this case, most of the metabolites exhibited a decrease in their centrality score with the exception of acetyl-CoA, one of the most central biomolecules in the whole metabolic network. Acetyl-CoA had a centrality score of zero in the disconnected case which becomes equal to 0.113 in the connected case (Figure 3B,C). The latter was approximately equal to half of the total sum of centrality scores for the pathway, meaning that even if only acetyl-CoA is significantly changed, this pathway will have an impact score of 0.5. In this dataset, the consideration of connectivity increased the impact score of “Glutathione metabolism” by almost seven times.

**Figure 5:**
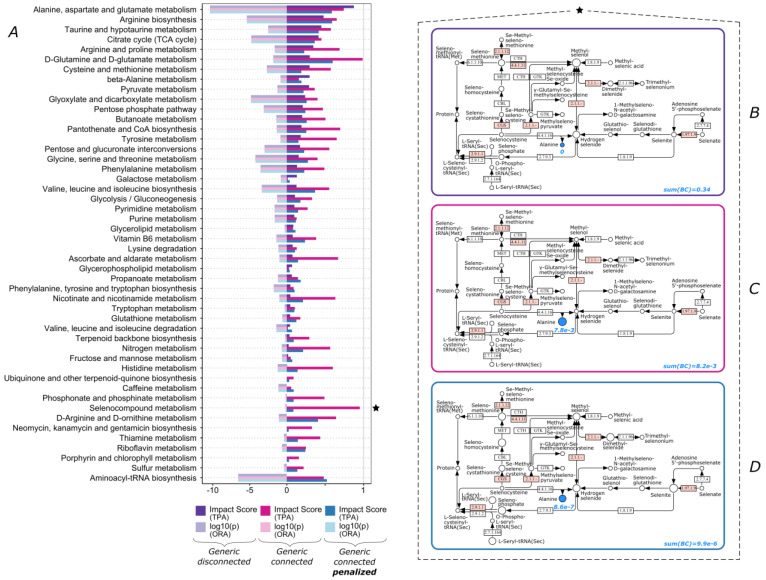
Pathway scoring comparison for dataset 4. (**A**) TPA impact scores and ORA log10(p) values for all pathways (pathways exhibiting zero values in all cases are omitted). ★ “Selenocompound metabolism” pathway for (**B**) generic disconnected, (**C**) generic connected, and (**D**) generic connected with penalization scheme. Blue color denotes significant compounds in this dataset, blue values are the computed betweenness centrality scores for these nodes, sum(BC) is the sum of betweenness centrality scores for all the nodes of the pathway, and node size corresponds to the relevant betweenness centrality scores of the nodes within the pathway. White and pink boxes denote human native and non-human native enzymatic reactions, respectively.

Similar to acetyl-CoA, amino acids are also very central to the metabolic network and can have similar effects on scoring. For dataset 4, the generic “Selenocompound metabolism” pathway exhibited a very high score of 0.95 when considering pathway connectivity (Figure 5A). When looking closer, we noticed that this was driven by a single metabolite, namely L-alanine (Figure 5C). Even though this is not a small pathway, alanine alone contributed 95% to the total sum of centrality scores. Especially since alanine is an end compound in this subnetwork and not as critical to selenocompound metabolism as other compounds, this score can be misleading.

We thus decided to implement a penalty scheme for very highly scoring nodes, or hubs, which normalizes node scores across each pathway (see Methods 2.5). When applying this scheme in the calculation, we observed that while the majority of pathways retained an impact score similar to their original score, the ones suffering from a hub-induced misrepresentation exhibited significantly lowered impact scores (Figure 5A). Concerning “Selenocompound metabolism”, its score was reduced to 0.09 for this dataset. In essence, this normalization acts as a filter in pathway scoring in order to remove potential outliers stemming from inherent network properties.

### 3.3. Over-Representation Analysis vs. Topological Pathway Analysis

In addition, we performed an ORA calculation for all the datasets (Figure 1, Figure 2, Figure 3, Figure 4 and Figure 5). Although the results of the two methods were in agreement for most pathways, there were some cases where only one method produced a high score. Since ORA does not take into account network topology, it is expected that it under- or over-scores a significantly perturbed pathway which could be captured more realistically by TPA. Some of the pathways that ORA deemed significant but had a zero TPA impact score were already discussed (Figure 1A—“Valine, leucine and isoleucine biosynthesis”; Figure 3A—“Linoleic acid metabolism” and “alpha-linolenic acid metabolism”). The opposite case was also encountered (Figure 5A—“Selenocompound metabolism”).

## 4. Discussion

As the fields of metabolomics and lipidomics grow rapidly, accurate interpretation of such data has become essential for understanding the functional outcome of metabolomics and lipidomics data. ORA and TPA are two of the most commonly used approaches for the functional interpretation of metabolomics data, however, the connectivity between different metabolic pathways and the inclusion of non-human native reactions have not been fully investigated. In this work, we investigated these two aspects of TPA and their effects on the outcome, using four publicly available metabolomics datasets. We used betweenness centrality as the node scoring method and KEGG as our pathway database, since they are the most commonly used choices in the community [15,34].

Although there has been a huge effort in improving the available databases, definition and size of pathways across databases are not harmonized. Thus, the selection of pathway database can affect the results of ORA and TPA analyses [1,49,50]. Even within a single pathway database, we observed that the choice between human-only and generic pathway definitions resulted in major differences in the outcome. Certain metabolic processes are not catalyzed by human native enzymes, thus, they are not included in the human-only definition of the metabolic process. To be able to capture these pathways, the generic approach is necessary. We would argue that for most cases the generic approach should be utilized, especially for untargeted metabolomics datasets. However, in studies where organism specificity is important, the organism-specific approach should be employed. 

In addition to pathway definitions, the decision regarding the treatment of individual pathways (i.e., connected or disconnected to each other) is critical. Non-topology methods such as ORA that rely on statistical evaluation are not affected by network connectivity; however, this is not the case for TPA. Although the consideration of each individual metabolic pathway (i.e., metabolic process) is helpful in the deconstruction of the metabolic network, this assumption of metabolic independence does not reflect the reality and could lead to incorrect outcomes. Even though both approaches are useful in the functional interpretation of metabolomics datasets, we believe that the connected consideration should be utilized for most cases, since all metabolic processes are interconnected. If the organism-specific pathway definition is used, connectivity is crucial to ensure that the number of isolated reactions is minimized. However, if any specific part of the metabolic network is of interest, a disconnected approach could be beneficial. In this case, a disconnected approach would reduce the effect of other pathways in terms of centrality scores. The pitfall of the connected approach is that very central biomolecules such as acetyl-CoA or amino acids will have such big scores that they will overshadow the majority of other metabolites in any pathway. To provide a solution for this problem, we have proposed a penalty scheme that effectively dampens the large node centrality scores, which correspond to hub compounds. By using this penalty approach, we ensure that the centrality scores within a pathway have comparable magnitudes, thus the scoring calculation remains unbiased. We believe such a normalization scheme in a connected network definition provides more realistic outcomes. In addition, alternative normalization approaches could be applied and evaluated.

Finally, it is important to note that ORA and TPA methods might lead to very different results in terms of pathway scoring. ORA does not account for network topology but the number of statistically significant compounds in each pathway will reflect on its output. Thus, both the connected and disconnected approaches would yield the same results. On the other hand, TPA puts emphasis on the topology, and the output might vary significantly depending on the pathway definitions and graph metrics. It is thus very important to have awareness of the advantages and limitations of each method, as well as being able to critically interpret their results. We believe that the combination of both methods would be advantageous. TPA should be used as the main tool to evaluate the magnitude of perturbation of each pathway, while ORA should be used as a statistical confirmation of the TPA outcome.

This study aims to raise awareness to the inherent pitfalls of TPA and provide some insight on various methodologies and strategies that should be considered before proceeding to the analysis of metabolomics datasets. Even though there can be no conclusive verdicts in the absence of a ground-truth dataset, we hope that our findings will spark more discussions and investigations in the community on the proper usage and interpretation of pathway analysis methodologies.

## Figures and Tables

**Table 1:** List of datasets used in this work.

Reference	Condition	Comparison	# of Statistically Significant Compounds	# of Significant Compounds Matched to KEGG IDs		ID of Dataset in This Work
				Manual Curation	Online Tool	
[35]	COVID-19	Non-severe vs. Healthy	474 *(p < 0.05)*	253	143	1a
		Non-COVID-19 vs. Healthy	272 *(p < 0.05)*	135	73	1b
[36]	Colorectal cancer	History of colorectal surgery vs. Healthy	81 *(p < 0.10)*	81 *	81 *	2
[37]	Hepatocellular carcinoma	Development risk factors	43 *(p < 0.05)*	31	25	3
[38]	Acute-on-chronic liver failure	Any stage markers vs. Acute decompensation	149 *(p < 0.05)*	131	120	4

An asterisk (*) denotes that KEGG IDs were provided by the authors with the corresponding dataset.

## Data Availability

Not applicable.

## Supplementary Materials

The following supporting information can be downloaded at: https://www.mdpi.com/article/10.3390/biom13020244/s1, Table S1: Datasets and matched compound identifiers used in the analysis.
